# Wideband Miniaturized Design of Complementary Spoof Surface Plasmon Polaritons Waveguide Based on Interdigital Structures

**DOI:** 10.1038/s41598-020-60244-7

**Published:** 2020-02-24

**Authors:** Bai Cao Pan, Guo Qing Luo, Zhen Liao, Jia Lin Cai, Ben Geng Cai

**Affiliations:** 1 College of Electromagnetics and Information Engineering, Hangzhou Dianzi University, Hangzhou, 310018 China; 2 State Key Laboratory of Millimetre Waves, School of Information Science and Engineering, Southeast University, Nanjing, 210096 China

**Keywords:** Electrical and electronic engineering, Electronic and spintronic devices

## Abstract

In this paper, we present to achieve a broadband miniaturized transmission waveguide based on complementary spoof surface plasmon polaritons (CSSPPs). For this purpose, a novel SSPP design that consists of a corrugated slot line and a group of additional interdigital structures (ISs) is proposed, which brings in an extra solution to control the cut-off property of CSSPPs. The transmission cut-off frequency of the proposed design decreases with the increasing of the number of the ISs. Since the width of CSSPP waveguide is directly related to the operating frequency, the degree of miniaturization can be modulated freely by carefully choosing the number of the ISs. A prototype of device with four-ISs introduced is designed and fabricated. And the cut-off frequency of the design decreases from 10 GHz to 5.3 GHz, when the ISs are added. Experimental results agree well with the numerical simulations. The proposed design illustrates great potentials in modern plasmonic integrated circuits.

## Introduction

Surface plasmon polaritons (SPPs) are classical surface modes excited along interface of metal and dielectric at visible and near-infrared wavelengths^1,2^. Such modes exhibit significant properties of near-field confinement, shorter operating wavelength and perfect low-pass transmission, which have been studied in a broad range of applications such as high-resolution imaging^3–6^, electromagnetically induced transparency (EIT)^7–9^, photovoltaic improvement and biosensing^10–13^.

However, in the longer-wavelength spectra, like microwave and terahertz waves, SPP mode cannot be excited directly, due to the perfect-electrical-conductor property of metal. And the traditional Sommerfield-Zenneck surface electromagnetic responses show weak field-confinement^14^. In order to take advantages of the significant properties of SPP modes in the lower frequencies in high-efficient and less-mutual-coupling applications, metamaterials are introduced to achieve novel surface responses that have similar dispersive properties with SPP modes – the spoof SPP (SSPP) mode. In 2004 and 2005, Pendry *et al*. and Hibbins *et al*. predicted theoretically and verified experimentally such SSPP surface mode on the surface with periodic subwavelength cubic holes^15,16^. Then a series of plasmonic designs have been reported to obtain the SSPP waves at both microwave and terahertz frequencies^17–20^. High-order SSPP mode along corrugated metallic strip was also studied to achieve extra working bands^21^. And spoof surface magnon polaritons was theoretically analyzed along corrugated PMC surface^22^.

In order to excite the SSPP mode efficiently, the dispersion properties related to geometry dimensions and field distribution in both homogeneous and inhomogeneous media are studied^23–25^. A series of matching approaches were reported to connect the SSPP waveguides and traditional transmission lines^26–30^. High-efficient transmissions of SSPP mode were experimental excited via co-planar waveguides, microstrip lines, and corrugated slot lines. Based on these designs, plenty of functional SSPP devices and circuits have been presented. Tunable multi-band and broadband filters were proposed by introducing additional metamaterial resonators^31–33^, coupling with oppositely oriented corrugated strips^34^ and cooperating with Substrate Integrated Waveguide (SIW)^35–37^. SSPP modes have been widely studied in applications of radiation, such as leaky-wave antennas^38^, end-fire radiation^39^, compact antenna designs^40^, and performance enhancement^41^. Broadband vortex beams carrying orbital angular momentum was reported by controlling the phase distribution of the unit cells^42,43^. Other kinds of devices of power dividers^44,45^, directional transmissions^46^, active excitations^47^ were also reported. With the development of integrated circuits, SSPP modes have become a research hotspot in forming plasmonic circuits. Multi-layer and multi-channel transmissions of SSPP have been proposed^30,48,49^. Co-planar devices have also been discussed in conformal applications^32,50^. Constructing miniaturized design of SSPP becomes an imperative requirement to meet challenges in modern plasmonic integrated circuits.

In this paper, we proposed a novel complementary SSPP (CSSPP) waveguide consisting of a corrugated slot line and a group of IS slabs inside the grooves. The corrugated slot line plays the key role in exciting the SSPP mode and the loaded IS array modulates the cut-off frequency into lower frequency band. The novel design shows properties of high efficiency and better field confinement. In addition, by changing the number of IS slabs, the cut-off property could be shifted in a wide range. A prototype with four IS slabs loaded in the grooves is proposed. The cut-off frequency decreases by 46% compared with that of the traditional CSSPP waveguide with the same widths. The miniaturized CSSPP waveguides show great potentials in the future plasmonic integrated circuits and systems.

## Miniaturized CSSPP Design

**Figure 1:**
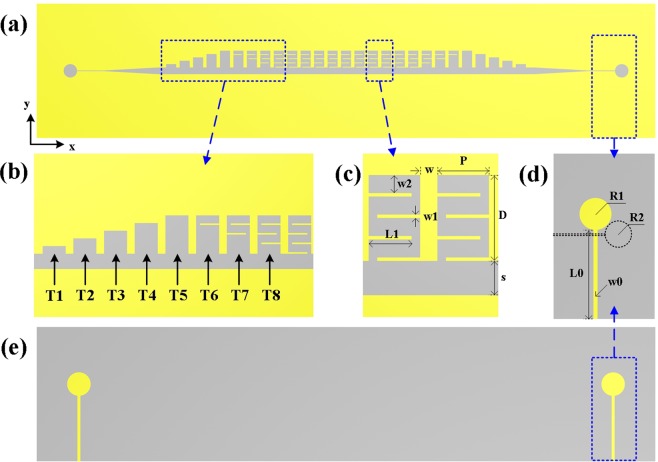
(**a**) Top and (**e**) back view of the schematic structure of the proposed design. Detailed view of (**b**) the eight-unit transition part, (**c**) two units of the novel waveguide and (**d**) feeding part.

The schematic structure of a IS-loaded CSSPP waveguide is shown in Fig. 1(a,e). The proposed design is excited via microstrip lines on the bottom, a gradient-varying slot and an eight-unit transition part. The transition part is illustrated detailed in Fig. 1(b). The first five cycles (T1-T5) are five groove structures with different depths for momentum matching. According to the dispersive properties of groove structures, gradually deepened grooves could obtain high efficient excitation of CSSPP mode. Cycles T6, T7 and T8 are grooves with the same depths and increasing numbers of IS slabs, during which further decreases of the cut-off frequencies could be obtained. The main transmission part of the proposed waveguide consists of a main slot and periodically grooves with four IS slabs. Two units of the novel waveguide are shown in Fig. 1(c) with dimensions labeled. The depth D, unit cycle P, and width w of the grooves are optimized to be 5 mm, 4 mm, and 1 mm, respectively. The width w1, length w2 of IS slab, and distance between IS slabs w2 are optimized to be 0.2 mm, 2.5 mm, and 1.25 mm, respectively. The width of the main slot s is 2 mm, and the substrate is F4B with thickness of 0.5 mm and a relative permittivity of 2.65. Designs of Vivaldi antenna in literatures provide good references to achieve excitation of slot lines. The back view of the feeding part is shown in Fig. 1(d), in which the black dashed line represents the slot and circular resonant cavity on the top layer. Signal propagates from the microstrip feeding line to the slot line via field coupling. The length and width of the feeding strip are L0 = 19.5 mm and w0 = 1.28 mm. The circular stub line with radius R1 = 7 mm at the end of the microstrip line improves the coupling efficiency and reduces the reflection. The circular cavity with radius R2 = 4.2 mm helps to improve coupling efficiency and realize unidirectional propagation in slot line. The circular cavity is connected to the main slot via a slot with width widening from 0.2 mm to 2 mm gradually.

**Figure 2:**
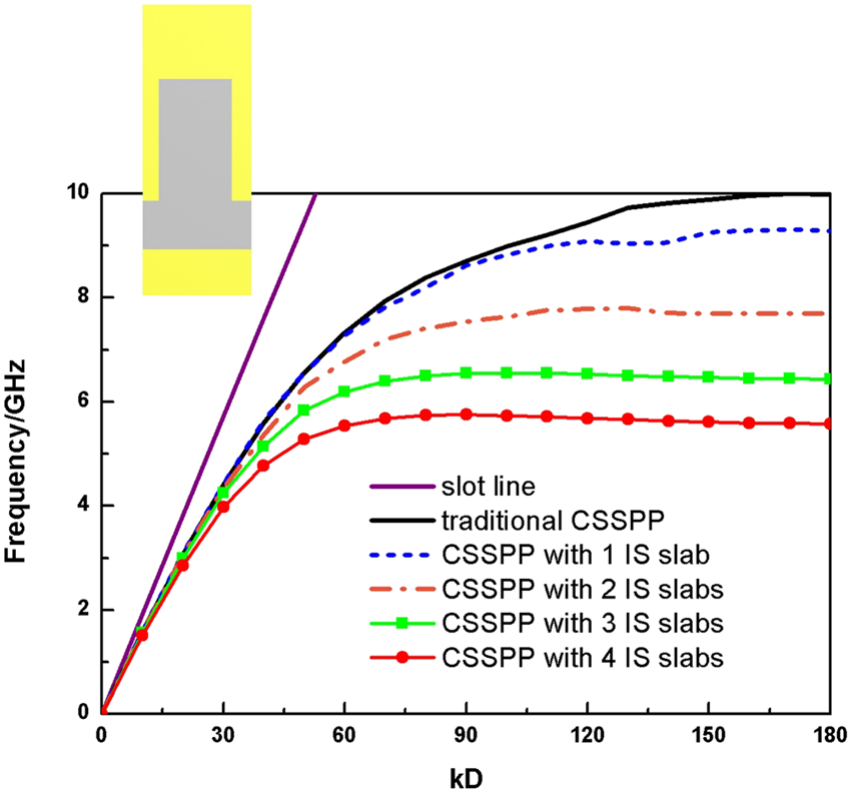
Dispersion properties of slot line (purple line), traditional CSSPP waveguide (black line) and novel design with one (blue dashed line), two (orange dash dot line), three (green line with squares) and four (red line with dots) IS slabs loaded. Inset: one unit of the traditional CSSPP.

As has been fully discussed in literatures^23,27^, the cutoff properties of the SSPP mode in homogeneous media are mainly determined by the depths of grooves. The bigger the depths are, the lower the cutoff frequencies would be. Therefore, to realize a lower operating frequency band usually requires a much larger dimension. The appearance of IS slabs enhances the capacitive resonance of the unit cell. The dispersion properties of the proposed structure performed on a homogeneous substrate are studied and illustrated in Fig. 2. The traditional corrugated CSSPP structure is compared with the proposed design with different numbers of IS slabs. One unit of the traditional CSSPP structure is shown in the inset of Fig. 2. The depths keep to be 5 mm, and the traditional CSSPP shows the cutoff property at 10 GHz. When IS slabs are introduced, we observe that the cutoff frequencies of the dispersion curves reduce gradually, which indicates that the momentum matching design of T6-T8 in the transition part is available.

Meanwhile, the dispersion curves of the IS-loaded design deviate more quickly from the curve of slot line, which means that it has a larger wave number. According to basic dispersive model of SPP,

$${k}_{0}^{2}={k}_{\parallel }^{2}+{k}_{\perp }^{2}$$

where *k*_0_, *k*_||_ and *k*_⊥_ are wave number in free space, along and perpendicular to the direction of propagation. For CSSPP mode, it always has relationship of *k*_||_ > *k*_0_. The larger the wave number *k*_||_ is, the larger the imaginary part of *k*_⊥_ would be. And this leads to faster attenuation of field intensity perpendicular to the direction of propagation. Hence, the near-field confinement is enhanced.

## Field Analysis and Measurement

**Figure 3:**
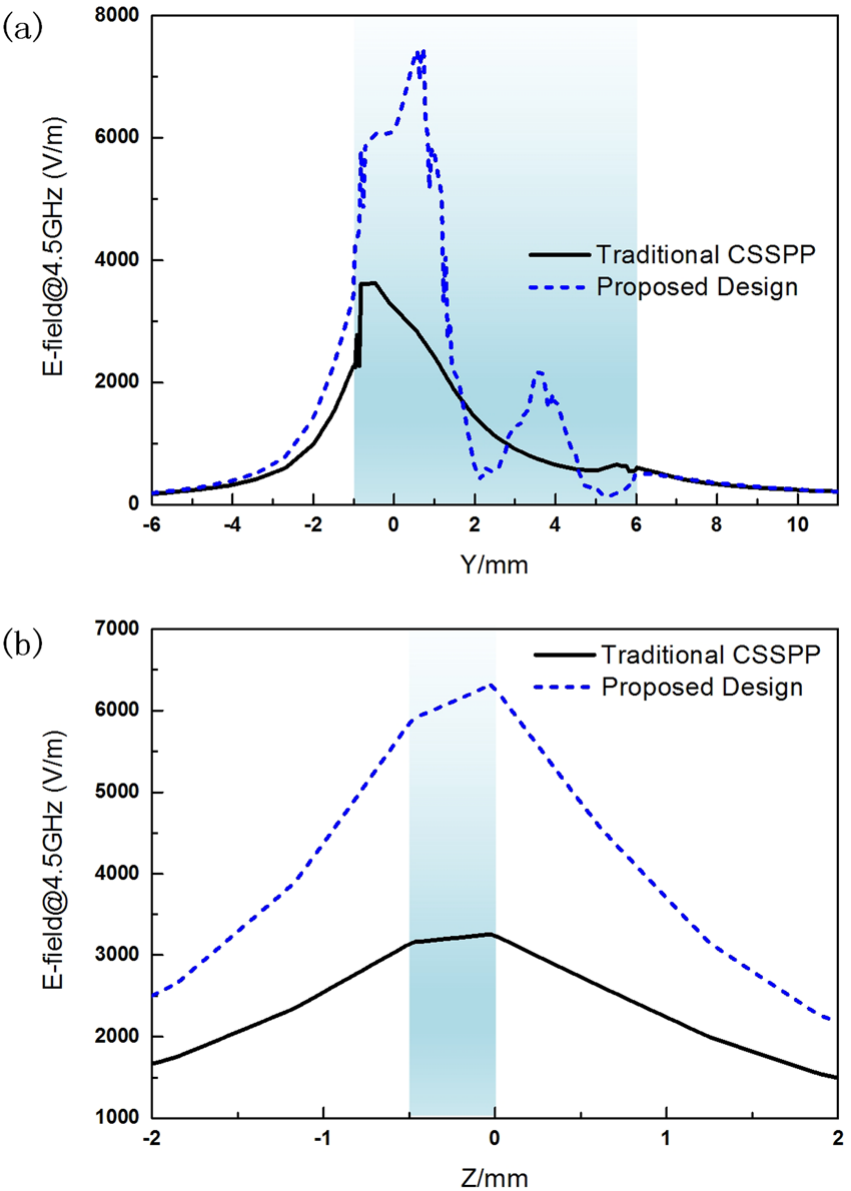
One-dimensional distribution of electric field along (**a**) curve 1 and (**b**) curve 2. The blue area marks the locations of the CSSPP structure (the main slot and IS-loaded grooves) and the substrate. (Curve 1: along y-direction from y = −6 mm to 11 mm at x = 0 mm & z = −0.2 mm; Curve 2: along z-direction from z = −2 mm to 2 mm at x = y = 0 mm).

**Figure 4:**
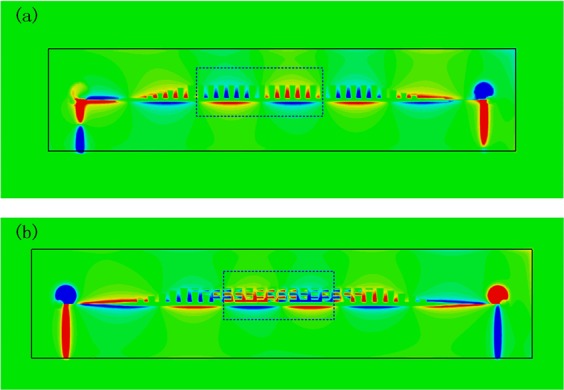
Two-dimensional distribution of electric field of (**a**) the traditional corrugated CSSPP waveguide and (**b**) the proposed design with IS-loaded. Areas within one phase period are labeled by blue dotted wire frame to illustrate the equivalent operating wavelength.

Figures 3 and 4 illustrate the simulated 1D and 2D near electric-field distributions to verify the high-performance of CSSPP mode. Near electric field intensity along two reference curves is studied. The coordinate origin is set at the center of the device. The design locates in x-y-plane and the top surface of the metal is set to be at position z = 0. For situation I, the reference curve is along y-direction (from y = −6 mm to y = 11 mm) and in the middle of the substrate in z-direction (z = −0.2 mm). The one-dimensional electric distribution along curve 1 is shown in Fig. 3(a). The blue area marks where the CSSPP structure locates. The positions of the main slot and groove structure are ${{\rm{y}}}_{1}\in [\,-\,1,1]$ and ${{\rm{y}}}_{2}\in [1,6]$, respectively. The black line and blue dashed line in the figure represent field intensity of traditional CSSPP waveguide and the proposed design. For situation II, the reference curve is along z-axis, from z = −2 mm to 2 mm. The one-dimensional electric distribution along curve 1 is shown in Fig. 3(b). The blue area marks where the substrate locates (${\rm{z}}\in [\,-\,0.5,0]$). From the figure, we can observe that the field is mainly enhanced inside the main slot area, and the proposed design possesses much stronger near field intensity. Thus, the proposed design shows improved near field enhancement.

Another important property of CSSPP mode is the shorter operating wavelength. For CSSPP mode, according to the dispersion properties, we could obtain the equivalent wavelength of the mode through relationship:

$$\beta =k\cdot D=\frac{2\pi \cdot D}{\lambda }$$

where D is the cycle of the periodic structures. From data in Fig. 2, we get the wavelength of the design at 4.5 GHz as ${\lambda }_{d,4.5G}$ = 38.9 mm. And the wavelength of the traditional CSSPP is ${\lambda }_{t,4.5G}$ = 46.5 mm. They are both much shorter than the wavelength in free space. Two-dimensional distributions of electric field are compared in Fig. 4 and areas within one phase cycle are labeled by dotted frame. High efficient transmissions are observed. For traditional waveguide, one phase cycle covers about 12 units. And one phase cycle of the proposed design covers about 10 units. The field distributions confirm our analysis of shorter wavelength.

**Figure 5:**
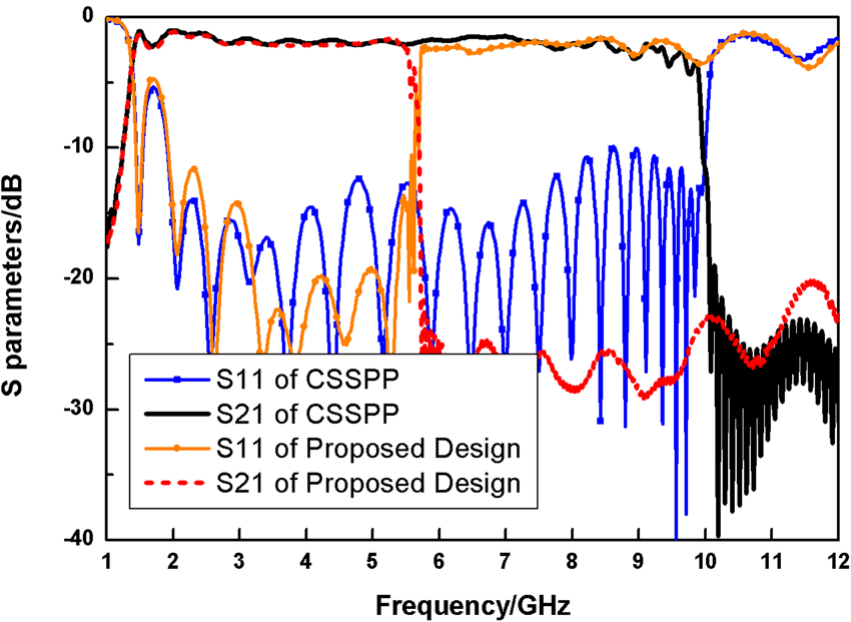
Comparison of simulated S parameters of the traditional CSSPP waveguide (blue line with squires and black line) and the proposed design (orange line with dots and red dashed line) with grooves’ depth of 5 mm.

Figure 5 shows the S-parameters of the traditional and novel CSSPP waveguides with depths of grooves to be 5 mm. The black solid line and the red dashed line represent transmission efficiencies of both situations, while blue line with squares and orange line with dots represent return losses. The excitation of the design does not support low frequency transmission. The cut-off properties around 1.5 GHz could be seen for both cases. And such cut-off frequencies are mainly restricted by the dimensions of the circular stub line and the circular cavity of the feeding parts. Meanwhile, significant reduction of the cutoff frequencies of CSSPP modes is observed. Moreover, the width of traditional CSSPP structure would enlarge to 10.6 mm to achieve a cutoff frequency of 5.3 GHz. The proposed design occupies less space to achieve a lower operating frequency band.

**Figure 6:**
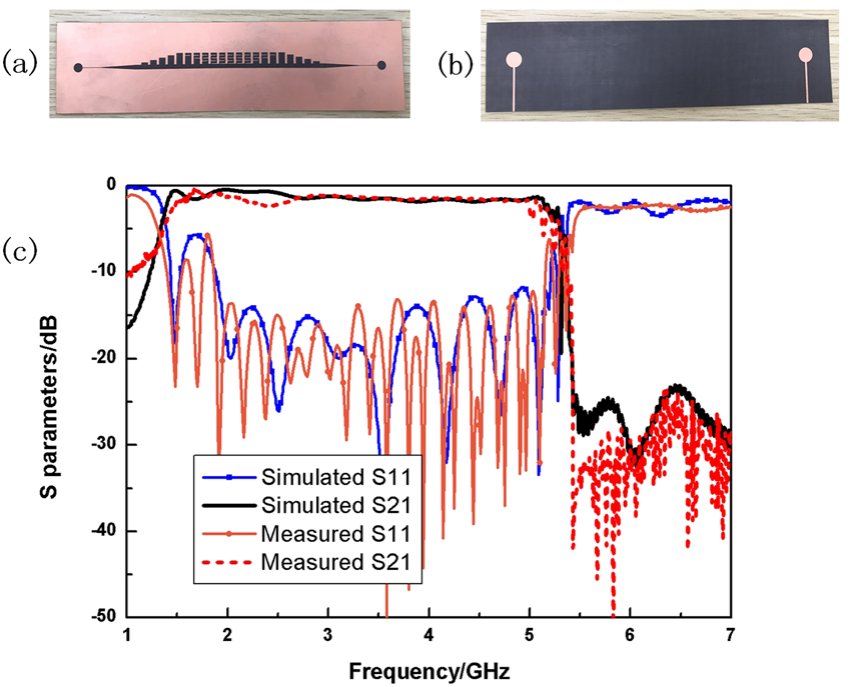
Photos of (**a**) top and (**b**) bottom of the proposed prototype. (**c**) Simulated (blue line with squires and black line) and measured (orange line with dots and red dashed line) S parameters of the proposed prototype.

The photos of the proposed compact CSSPP waveguide are illustrated in Fig. 6(a,b). From the dispersion properties shown in Fig. 2, we can surely conclude that, the more the IS slabs there are, the lower the cut-off frequency would be. Namely a better miniaturization property could be achieved. However, the space in each groove is limited, and the increase of IS slabs leads to undesired difficulties in matching designs. Therefore, a scheme of four IS slabs is chosen for sample. In the experiments, the Agilent Vector Network is used to measure the S scattering parameters via coaxial lines and SMA connectors. The simulated and measured transmissions (black line and red dashed line) and reflections (blue line with squares and orange line with dots) are compared in Fig. 6(c). A traditional CSSPP transmission could be seen and the measured results agree very well with the numerical simulations. It’s noteworthy that the area of the metallic surface in the sample is much larger than the slot area. In certain applications, it’s feasible to narrow the width of the metallic surface. The transmissions would keep efficient as long as the distances between outer edges and the corrugated slot are not too small. Meanwhile, according to the high-confinement property of CSSPP mode, such design could also be used in conformal applications, even for relatively sharp transitions.

## Conclusion

A novel CSSPP waveguide consisting of a corrugated slot line loaded with IS slabs is proposed to achieve lower operating frequency band with smaller widths. It has been shown that the novel design has have better ability of near field enhancement and shorter operating wavelength, compared with the traditional CSSPP structures. A prototype with four IS slabs loaded is demonstrated. The cutoff frequency drops to 5.3 GHz without occupying extra space. The miniaturized transmission design could work as the basis of coplanar plasmonic circuits, showing great potentials in the applications of complex and compact plasmonic systems.

## Methods

The commercial software, CST Microwave Studio, were used to simulate the transmission, power distribution and return loss of the proposed miniaturized CSSPP waveguide. Eigen-mode calculation was performed to studied the dispersion properties of the unit structures. The sample was fabricated on printed circuit board of F4B, with relative permittivity of 2.65 and loss tangent of 0.001. In experiments, both terminals of the design were connected to the Agilent vector network analyzer via coaxial cables to measure transmission and reflection.

## References

[1] Maier, S. A. Plasmonics: Fundamentals and Applications, Springer Science & Business Media, NY, USA (2007).

[2] Törmä P, Barne WL (2014). Strong coupling between surface plasmon polaritons and emitters: a review. Reports on Progress in Physics.

[3] Li JF, Li CY, Aroca RF (2017). Plasmon-enhanced fluorescence spectroscopy. Chemical Society Reviews.

[4] Willets KA (2017). Super-resolution imaging and plasmonics. Chemical Reviews.

[5] Chien FC, Lin CY, Abrigo G (2018). Enhancing the blinking fluorescence of single-molecule localization imaging by using a surface-plasmon-polariton-enhanced substrate. Physical Chemistry Chemical Physics.

[6] Bezryadina A (2018). High spatiotemporal resolution imaging with localized plasmonic structured illumination microscopy. ACS Nano.

[7] Liao Z (2016). Electromagnetically induced transparency metamaterial based on spoof localized surface plasmons at terahertz frequencies. Scientific Reports.

[8] Xia SX (2018). Plasmonically induced transparency in double-layered graphene nanoribbons. Photonics Research.

[9] Lu H (2018). Flexibly tunable high-quality-factor induced transparency in plasmonic systems. Scientific Reports.

[10] Bauch M (2014). Plasmon-enhanced fluorescence biosensors: a review. Plasmonics.

[11] Rodrigo D (2015). Mid-infrared plasmonic biosensing with graphene. Science.

[12] Nguyen H (2015). Surface plasmon resonance: a versatile technique for biosensor applications. Sensors.

[13] Masson JF (2017). Surface plasmon resonance clinical biosensors for medical diagnostics. ACS Sensors.

[14] Yu N (2010). Designer spoof surface plasmon structures collimate terahertz laser beams. Nature Materials.

[15] Pendry JB, Martin-Moreno L, Garcia-Vidal FJ (2004). Mimicking surface plasmons with structured surfaces. Science.

[16] Hibbins AP, Evans BR, Sambles JR (2005). Experimental verification of designer surface plasmons. Science.

[17] Navarro-Cía M (2009). Broadband spoof plasmons and subwavelength electromagnetic energy confinement on ultrathin metafilms. Optics Express.

[18] Williams CR (2008). Highly confined guiding of terahertz surface plasmon polaritons on structured metal surfaces. Nature Photonics.

[19] Tang HH, Ma TJ, Liu PK (2016). Experimental demonstration of ultra-wideband and high-efficiency terahertz spoof surface plasmon polaritons coupler. Applied Physics Letters.

[20] Shen X (2013). Conformal surface plasmons propagating on ultrathin and flexible films. Proceedings of the National Academy of Sciences.

[21] Liu X (2013). High-order modes of spoof surface plasmonic wave transmission on thin metal film structure. Optics Express.

[22] Liu L (2014). A corrugated perfect magnetic conductor surface supporting spoof surface magnon polaritons. Optics Express.

[23] Liu YQ (2016). Spoof surface plasmon modes on doubly corrugated metal surfaces at terahertz frequencies. Journal of Physics D: Applied Physics.

[24] Gric T, Cada M (2014). Analytic solution to field distribution in one-dimensional inhomogeneous media. Optics Communications.

[25] Gric T (2015). Analytic solution to field distribution in two-dimensional inhomogeneous waveguides. Journal of Electromagnetic Waves and Applications.

[26] Gao X (2013). Ultrathin dual-band surface plasmonic polariton waveguide and frequency splitter in microwave frequencies. Applied Physics Letters.

[27] Ma HF (2014). Broadband and high-efficiency conversion from guided waves to spoof surface plasmon polaritons. Laser & Photonics Reviews.

[28] Kianinejad A, Chen ZN, Qiu CW (2015). Design and modeling of spoof surface plasmon modes-based microwave slow-wave transmission line. IEEE Transactions on Microwave Theory and Techniques.

[29] Kianinejad A, Chen ZN, Qiu CW (2016). Low-loss spoof surface plasmon slow-wave transmission lines with compact transition and high isolation. IEEE Transactions on Microwave Theory and Techniques.

[30] Pan BC (2016). Multi-layer topological transmissions of spoof surface plasmon polaritons. Scientific Reports.

[31] Pan BC (2014). Controlling rejections of spoof surface plasmon polaritons using metamaterial particles. Optics Express.

[32] Zhou YJ, Xiao QX (2017). Electronically controlled rejections of spoof surface plasmons polaritons. Journal of Applied Physics.

[33] Jaiswal RK, Pandit N, Pathak NP (2018). Spoof Surface Plasmon Polaritons Based Reconfigurable Band-Pass Filter. IEEE Photonics Technology Letters.

[34] Yin JY (2015). Broadband frequency-selective spoof surface plasmon polaritons on ultrathin metallic structure. Scientific Reports.

[35] Guan DF (2017). Hybrid spoof surface plasmon polariton and substrate integrated waveguide transmission line and its application in filter. IEEE Transactions on Microwave Theory and Techniques.

[36] Chen P (2018). Hybrid Spoof Surface Plasmon Polariton and Substrate Integrated Waveguide Broadband Bandpass Filter With Wide Out-of-Band Rejection. IEEE Microwave and Wireless Components Letters.

[37] Yang ZB (2018). Low-Loss Spoof Surface Plasmon Polariton Based on Folded Substrate Integrated Waveguide. IEEE Antennas and Wireless Propagation Letters.

[38] Zhang QL, Zhang Q, Chen Y (2017). Spoof surface plasmon polariton leaky-wave antennas using periodically loaded patches above PEC and AMC ground planes. IEEE Antennas and Wireless Propagation Letters.

[39] Kandwal A (2017). Low-profile spoof surface plasmon polaritons traveling-wave antenna for near-endfire radiation. IEEE Antennas and Wireless Propagation Letters.

[40] Yang Y (2018). Miniaturized High-Order-Mode Dipole Antennas Based on Spoof Surface Plasmon Polaritons. IEEE Antennas and Wireless Propagation Letters.

[41] Zhang XF, Fan J, Chen JX (2018). High Gain and High-Efficiency Millimeter-Wave Antenna Based on Spoof Surface Plasmon Polaritons. IEEE Transactions on Antennas and Propagation.

[42] Wang, H. *et al*. Spin-to-Orbital Angular Momentum Conversion with Quasi-Continuous Spatial Phase Response. *Advanced Optical Materials*, 19001188 (2019).

[43] Wang H (2019). Vortex beam generated by circular-polarized metasurface reflector antenna. Journal of Physics D: Applied Physics.

[44] Wu Y (2016). Single-conductor co-planar quasi-symmetry unequal power divider based on spoof surface plasmon polaritons of bow-tie cells. AIP Advances.

[45] Zhou SY (2019). Four-Way Spoof Surface Plasmon Polaritons Splitter/Combiner. IEEE Microwave and Wireless Components Letters.

[46] Xu Y (2013). Broadband asymmetric waveguiding of light without polarization limitations. Nature Communications.

[47] Zhang HC (2015). Second-harmonic generation of spoof surface plasmon polaritons using nonlinear plasmonic metamaterials. Acs Photonics.

[48] Liu L (2014). Multi-channel composite spoof surface plasmon polaritons propagating along periodically corrugated metallic thin films. Journal of Applied Physics.

[49] Pan BC, Zhang HC, Cui TJ (2017). Multilayer transmissions of spoof surface Plasmon polaritons for multifunctional applications. Advanced Materials Technologies.

[50] Xu J (2016). Low-pass plasmonic filter and its miniaturization based on spoof surface plasmon polaritons. Optics Communications.

